# The NiFe Hydrogenases of the Tetrachloroethene-Respiring Epsilonproteobacterium *Sulfurospirillum multivorans*: Biochemical Studies and Transcription Analysis

**DOI:** 10.3389/fmicb.2017.00444

**Published:** 2017-03-20

**Authors:** Stefan Kruse, Tobias Goris, Maria Wolf, Xi Wei, Gabriele Diekert

**Affiliations:** ^1^Department of Applied and Ecological Microbiology, Institute of Microbiology Friedrich Schiller University, Germany; ^2^Dianovis GmbHGreiz, Germany; ^3^Department Isotope Biogeochemistry, Helmholtz Centre for Environmental Research-UFZLeipzig, Germany; ^4^YMC Europe GmbHDinslaken, Germany

**Keywords:** organohalide respiration, hydrogenase, real-time PCR, regulation of gene expression, hydrogen, anaerobic respiration

## Abstract

The organohalide-respiring Epsilonproteobacterium *Sulfurospirillum multivorans* is able to grow with hydrogen as electron donor and with tetrachloroethene (PCE) as electron acceptor; PCE is reductively dechlorinated to *cis*-1,2-dichloroethene. Recently, a genomic survey revealed the presence of four gene clusters encoding NiFe hydrogenases in its genome, one of which is presumably periplasmic and membrane-bound (MBH), whereas the remaining three are cytoplasmic. To explore the role and regulation of the four hydrogenases, quantitative real-time PCR and biochemical studies were performed with *S. multivorans* cells grown under different growth conditions. The large subunit genes of the MBH and of a cytoplasmic group 4 hydrogenase, which is assumed to be membrane-associated, show high transcript levels under nearly all growth conditions tested, pointing toward a constitutive expression in *S. multivorans*. The gene transcripts encoding the large subunits of the other two hydrogenases were either not detected at all or only present at very low amounts. The presence of MBH under all growth conditions tested, even with oxygen as electron acceptor under microoxic conditions, indicates that MBH gene transcription is not regulated in contrast to other facultative hydrogen-oxidizing bacteria. The MBH showed quinone-reactivity and a characteristic UV/VIS spectrum implying a cytochrome *b* as membrane-integral subunit. Cell extracts of *S. multivorans* were subjected to native polyacrylamide gel electrophoresis (PAGE) and hydrogen oxidizing activity was tested by native staining. Only one band was detected at about 270 kDa in the particulate fraction of the extracts, indicating that there is only one hydrogen-oxidizing enzyme present in *S. multivorans*. An enrichment of this enzyme and SDS PAGE revealed a subunit composition corresponding to that of the MBH. From these findings we conclude that the MBH is the electron-donating enzyme system in the PCE respiratory chain. The roles for the other three hydrogenases remain unproven. The group 4 hydrogenase might be involved in hydrogen production upon fermentative growth.

## Introduction

Molecular hydrogen (H_2_) is one of the primary electron donors for many anaerobic respiratory processes mediated by prokaryotes. Organohalide respiration, in which energy conservation is coupled to the reductive dehalogenation of halogenated organic compounds, is no exception in this regard. Obligate organohalide-respiring bacteria such as *Dehalococcoides mccartyi* and *Dehalobacter* *restrictus* rely solely on H_2_ as electron donor (Holliger et al., 1998; Löffler et al., 2013), while most versatile organohalide-respiring organisms, e.g., *Desulfitobacterium* spp. or *Sulfurospirillum* spp., use H_2_ as one of many electron donors (Scholz-Muramatsu et al., 1995; Luijten et al., 2003; Villemur et al., 2006; Goris and Diekert, 2016). One feature, which is common to many genomes of organohalide-respiring bacteria, is the presence of multiple hydrogenase-encoding gene clusters (Kube et al., 2005; Seshadri et al., 2005; Nonaka et al., 2006; Kruse et al., 2013, 2015; Goris et al., 2014). Besides H_2_ oxidation in respiratory processes, which is usually mediated by a membrane-bound NiFe hydrogenase, several different cellular metabolic processes are thought to recruit the other hydrogenase enzymes for either H_2_ oxidation or H^+^ reduction. The roles of these hydrogenases are unknown and under debate (Seshadri et al., 2005; Rupakula et al., 2013; Goris et al., 2014; Mansfeldt et al., 2014; Kruse et al., 2015). For example, cytoplasmic hydrogenases with an NAD(P)^+^ binding motif might be responsible for generating reducing equivalents for biosynthetic pathways or for balancing the cellular redox state. Group 4 Ech-type hydrogenases, which often harbor several large membrane-integral subunits, might also be involved in energy-conserving processes, as reported for methanogens (Hedderich and Forzi, 2005; Welte et al., 2010).

**FIGURE 1:**
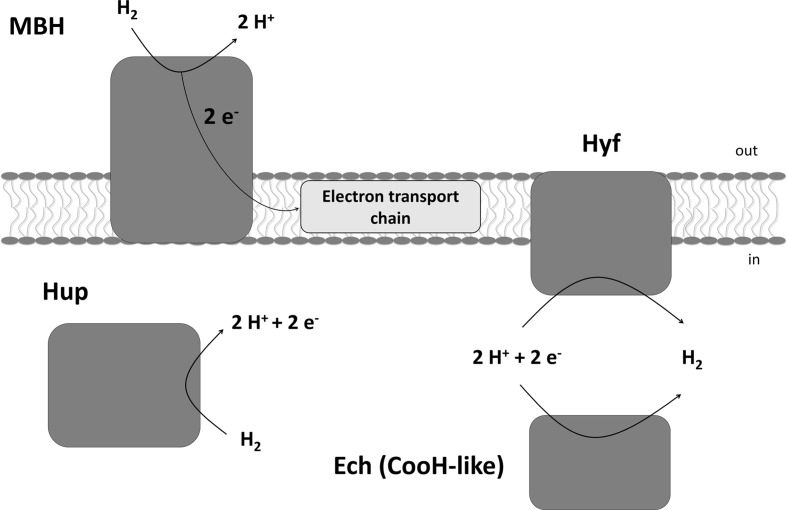
**Putative physiological roles of the four hydrogenases of *Sulfurospirillum multivorans* and their subcellular localization.** MBH, membrane-bound hydrogenase HydABC; Hup, cytoplasmic uptake hydrogenase HupSL; Hyf, HyfABCEFGHI similar to *E. coli* hydrogenase 4; Ech, EchEDFC, similar to the CO-induced hydrogenase of *Carboxydothermus hydrogenoformans*, but in *S. multivorans* lacking any membrane subunit.

*Sulfurospirillum multivorans* is a versatile organohalide-respiring Epsilonproteobacterium that uses H_2_ or other compounds such as formate or pyruvate as electron donors and chlorinated ethenes or, e.g., nitrate or fumarate as electron acceptor (Scholz-Muramatsu et al., 1995; Goris and Diekert, 2016). Recently, it has also been shown that the organism is able to utilize O_2_ as terminal electron acceptor under microoxic conditions with about 5% O_2_ in the gas phase (Goris et al., 2014). The genome of *S. multivorans* contains gene clusters encoding four different NiFe hydrogenases. One is predicted to be a periplasmic, membrane-bound H_2_-oxidizing enzyme (membrane-bound hydrogenase, MBH, HydABC encoded by SMUL_1423-1425), which is very similar (50 to 76% subunit amino acid sequence identity) to the characterized uptake hydrogenase of *Wolinella succinogenes* (Dross et al., 1992). The small subunit of the MBH of both organisms, HydA, contains a TAT signal peptide which is cleaved off after maturation and transport of the MBH into the periplasm. HydC is a membrane-integral cytochrome *b* subunit which connects the hydrogenase to the quinone pool (Gross et al., 2004). The other three hydrogenases contain no signal peptide motif and are therefore considered to be cytoplasmic (Goris et al., 2014). One of these three enzymes (HupSL, encoded by SMUL_1421-1422) is related to cytoplasmic H_2_-consuming hydrogenases and regulatory hydrogenases, the other two can be classified as group 4 hydrogenases, which are known to produce H_2_ (Vignais and Billoud, 2007). The hydrogenase encoded by the *ech* gene cluster (structural proteins EchEDFC encoded by SMUL_1307-1310) bears similarities to the CO-induced hydrogenase of *Carboxydothermus hydrogenoformans* (Soboh et al., 2002), but the *S. multivorans* genome does not contain genes coding for a CO dehydrogenase. Hydrogenase membrane subunit genes were not found on the *ech* gene cluster, which is remarkable, since all group 4 hydrogenase gene clusters normally contain genes encoding membrane-integral subunits (Greening et al., 2016). The fourth hydrogenase comprises eight subunits (HyfABCEFGHI encoded by SMUL_2383-2390), of which four are predicted to be membrane-integral. This hydrogenase is similar to hydrogenase 4 (Hyf) of *Escherichia coli*. This enzyme found in several Enterobacteriaceae might interact with a cytoplasmic formate dehydrogenase (FdhF) to form a formate hydrogen lyase (FHL) complex (Trchounian et al., 2012; Sargent, 2016). At least two of the hydrogenases of *S. multivorans*, the MBH and the Hyf, are produced during tetrachloroethene (PCE) respiration with either pyruvate or formate as sole electron donor (Goris et al., 2015). The periplasmically oriented MBH is assumed to be the main H_2_-oxidizing enzyme in *S. multivorans*, since H_2_-oxidizing activity was detected in whole cells and the majority of this activity was found to be membrane-associated (Miller et al., 1996). However, these assays were performed with cells and extracts from *S. multivorans* grown with H_2_/fumarate, while a study with H_2_/PCE-grown cells was never carried out. Deduced from amino acid sequence similarity, HupSL was discussed to play a role in either the recycling of H_2_ produced cytoplasmically (e.g., during N_2_ fixation) or to deliver low-potential reducing equivalents for anabolic purposes comparable to *Aquifex aeolicus* hydrogenase III (Guiral et al., 2005). However, it could also be involved in H_2_-dependent PCE respiration. The two group 4 hydrogenases might play a role in H_2_ production from excess reducing equivalents during fermentative growth. The putative roles of the four hydrogenases in *S. multivorans* are depicted in **Figure 1**.

Usually hydrogenase expression underlies specific regulation depending on their physiological role and growth conditions (Kovács et al., 2005; Greening and Cook, 2014). For example, uptake hydrogenases gene transcription is upregulated when H_2_ is available, NiFe hydrogenase gene expression is down-regulated when the cofactor nickel is absent, and O_2_-sensitive hydrogenases are not expressed under oxic conditions. However, in both Epsilonproteobacteria and organohalide-respiring bacteria nearly nothing is known about hydrogenase gene regulation. In this study we wanted to (1) reveal the hydrogen uptake metabolism of *S. multivorans* during H_2_-driven PCE respiration and characterize the uptake hydrogenase involved in this process and (2) investigate the transcript profiles of hydrogenase catalytic subunit genes during different growth conditions, which might give information about the regulation of the four hydrogenases and their physiological role in *S. multivorans*.

## Experimental Procedures

### Cultivation of *S. multivorans*

*Sulfurospirillum multivorans* (DSMZ 12446) was grown anaerobically at 28°C in rubber stoppered glass bottles shaken at 150 rpm with an aqueous to gas phase ratio of 1:1. The medium was described previously containing cysteine as reductant but did not contain vitamin B_12_ (cyanocobalamin) here (Scholz-Muramatsu et al., 1995). Before autoclaving, the gas phase contained 150 kPa N_2_. Pyruvate (40 mM) was used as electron donor and fumarate (40 mM), PCE, nitrate (10 mM), or 5% O_2_ as electron acceptor. In the latter case, O_2_ concentration in the gas phase was measured with the Microx 4 oxygen meter (PreSens Precision Sensing GmbH, Regensburg, Germany). The amount of 5% O_2_ in the gas phase corresponded to approximately 0.5 mg/L medium in the liquid phase. Cells growing with pyruvate as electron donor were cultivated at least 50 transfers without H_2_ in the gas phase to test for possible long-term regulation effects. PCE was added to the medium in a nominal concentration of 10 mM from a 0.5 M PCE hexadecane stock solution. When cells were cultivated with H_2_ as electron donor, a gas phase of H_2_ (150 kPa) was applied and acetate (5 mM) was added as carbon source. For experiments with N_2_ as sole nitrogen source, ammonium chloride was omitted from the basal medium and N_2_ (150 kPa) was used as gas phase. The following energy substrate combinations were used: pyruvate/fumarate, pyruvate/PCE, pyruvate/O_2_, pyruvate alone, H_2_/PCE, and H_2_/nitrate. Growth was monitored photometrically at 578 nm.

### Cell Harvesting and Samples Preparation

*Sulfurospirillum multivorans* cells were harvested oxically in the mid-exponential growth phase by centrifugation (12,000 × *g*, 10 min at 10°C). Resulting cell pellets were washed twice in 50 mM Tris-HCl (pH 7.5) and resuspended in two volumes (2 ml per g cells) of the same buffer with additional DNase I (AppliChem, Darmstadt, Germany) and protease inhibitor (one tablet for 10 ml buffer; cOmplete Mini, EDTA-free; Roche, Mannheim, Germany). Cell disruption was carried out semi-anoxically using a French Press cell (1500 psi), which was made anoxic in a glovebox (Coy Laboratory Products Inc., Grass Lake, MI, USA), while the disruption itself was performed outside the tent. The obtained crude extracts were used for biochemical experiments. Cells were fractionated by ultracentrifugation (36,000 × *g*, 45 min at 4°C), supernatants were designated soluble fractions (SF). The pellets were resuspended in two volumes (2 ml per g membrane pellet) 50 mM Tris-HCl (pH 7.5); a part of this suspension was used further as membrane fraction (MF), while the rest was mixed with 0.1% Triton X-100 and stirred at 4°C overnight. Solubilized membrane extract (ME) was obtained from the resulting supernatant after ultracentrifugation (36,000 × *g*, 45 min at 4°C).

### Enrichment of the Membrane-Bound Hydrogenase

Enrichment was carried out at room temperature in an anoxic chamber with an atmosphere of 98% N_2_ and 2% H_2_ (Coy Laboratory Products Inc., Grass Lake, MI, USA) using an ÄKTA-FPLC system (GE Healthcare Europe GmbH, Freiburg, Germany). Cells were grown in 2 L Schott bottles (aqueous to gas phase ratio 1:1) with pyruvate and fumarate. Harvesting, disruption and fractionation was done as described in the previous chapter, except that 1% digitonin was used as detergent. The filtered solubilized ME was fractionated via an anion exchange column (Q-Sepharose HP column 10/10, GE Healthcare Europe GmbH, Freiburg, Germany). The Q-Sepharose column was pre-equilibrated with 50 mM Tris-HCl (pH 8.0), 0.5 mM DTT and 0.1% (v/v) Triton X-100. Subsequently, the column was washed with 200 ml of the same buffer. Elution of proteins was achieved with a linear salt gradient from 0 to 0.5 M KCl at a flow rate of 2 ml/min. Fractions containing hydrogenase activity eluted at approximately 0.2 M KCl. H_2_-oxidizing activity was checked photometrically and by activity stained blue native (BN) polyacrylamide gel electrophoresis (PAGE), while purity was checked by SDS PAGE and silver-stained BN PAGE.

### Blue Native Gel Activity Staining PAGE and SDS PAGE

Non-denaturing PAGE was performed with native gels from SERVA (SERVA*GEL* N, Heidelberg, Germany). The anaerobic running buffer system was composed of a cathode buffer (50 mM Tricine, 15 mM BisTris-HCl, 1 ml/L Serva Blue G; pH 7.0) and anode buffer (15 mM BisTris-HCl; pH 7.0). Samples were mixed with twofold loading dye (1 M 6-Aminocaproic acid; 100 mM BisTris-HCl pH 7.0; 100 mM NaCl; 20% glycerol; 0.1% Serva Blue G250). The protein marker used was a SERVA Native Marker Liquid Mix. The gels were run in a vertical polyacrylamide electrophoresis system (Minigel-Twin, Biometra GmbH, Göttingen, Germany) starting with 50 V for 10 min and subsequently 200 V for about 2 h. The gel electrophoresis was performed in an anoxic glovebox (Coy Laboratory Products Inc., Grass Lake, MI, USA). H_2_-oxidizing activity was visualized as follows: The gel was transferred to an anoxic Schott bottle filled with 50 mM Tris-HCl (pH 8.0) buffer containing 1 mM benzyl viologen (BV) as primary electron acceptor and redox mediator and 1 mM triphenyl tetrazolium chloride (TTC) as terminal electron acceptor for permanent staining. The bottle was then flushed with pure H_2_ for 30 min. The incubation was carried out in a 28°C water bath until first bands were visible and then stopped by removing the native gel from the bottle. Fractions obtained from enrichment procedure were subjected to SDS-PAGE, which was silver stained after the run.

### Photometric Measurement of Hydrogenase Activity

H_2_-oxidizing activity was measured spectrophotometrically in butyl rubber stoppered glass cuvettes filled with 1 ml H_2_-saturated buffer (50 mM Tris-HCl, pH 8.0) made anoxic by flushing with H_2_ for 5 min. Redox dyes used were 1 mM benzyl viologen (BV), 1 mM methyl viologen (MV), or 1 mM methylene blue (MB) at 30°C. The colorization caused by the reduction of BV or MV was recorded at 578 nm and decolorization of MB was monitored at 570 nm. Additionally, activity was measured with the menaquinone analogs 1,4-naphthoquinone and 2,3-dimethyl-1,4-naphthoquinone (DMN). Changes in absorption during reduction of these derivatives were recorded at 270 nm. The anoxic reaction buffer contained 0.2 mM DMN or 1,4-naphthoquinone in 50 mM glycylglycine (pH 8.0) and 0.5 mM DTT. The assay was performed in rubber-stoppered quartz cuvettes, flushed with H_2_. Reduction of DMN was recorded using the absorbance difference at 270 and 290 nm. Determination of protein concentration was done according to the method of Bradford (1976). Activity values are given in nanokatal (oxidation of 1 nmol H_2_ per second). Temperature and pH dependance was measured with enriched hydrogenase using a 50 mM Britton-Robinson buffer system (50 mM H_3_BO_3_, 50 mM H_3_PO_4_, 50 mM acetate) in a pH range from 5.5 to 10 and a temperature range between 10 and 50°C.

### Isolation of RNA, Reverse Transcription, and Quantitative Real-Time PCR

Isolation of total RNA from *S. multivorans* was done using the RNeasy minikit (Qiagen, Hilden, Germany). Remaining genomic DNA was removed with DNase I (RNase free; Roche, Mannheim, Germany). For quality check of the RNA, agarose gel electrophoresis was performed. For all quantitative real-time PCR (qPCR) experiments, the RNA was isolated in the mid-exponential growth phase of three independently grown cultures. For cDNA synthesis, 1 μg of RNA was used as starting material in the RevertAid First Strand cDNA Synthesis kit (Thermo Scientific, Schwerte, Germany). The reaction mixture contained 1 μg RNA, 2.5 μl reverse primer, 3.5 μl 5x reaction buffer, and 2 μl 10 mM dNTP mix. It was filled up to a final volume of 17.5 μl with PCR-grade water (Fermentas, St. Leon Rot, Germany). To 10.5 μl of the mix, 0.5 μl RevertAid Reverse transcriptase (RT; 200 U/μl) was added, the residual amount (mix without RT) was used as negative control. The mix was incubated for 1 h at 42°C in a thermo cycler (Mastercycler, Personal, Eppendorf, Hamburg, Germany) and the reaction was stopped at 72°C for 5 min. Transcript levels of the different genes were compared by qPCR with the Maxima SYBR green qPCR master mix (Fermentas, St. Leon Rot, Germany); the primer pairs used are listed in Supplementary Table 1. The assay was performed in triplicates in a CFX96 qPCR machine (Bio-Rad, Munich, Germany). The PCR reaction mixture included 2.5 μl cDNA, 0.5 μM of each primer, and 6 μl 2x Maxima SYBR green qPCR master mix and was filled up to a final volume of 12 μl with PCR-grade water. Two negative controls were used, one with water as qPCR template and a reverse transcriptase negative control for each tested gene. Melting curve analysis was performed to exclude the formation of primer dimers or unspecific byproducts. Before performing the qPCR experiments, primer efficiency was tested for all primer pairs used in this study to ensure accurate and comparable amplification (see Supplementary Data Sheet 2). Three different control genes for normalization were tested: 16S rRNA, *recA*, *rpoB*. Only the first was useful as control gene under all growth conditions applied. Obtained data were thus normalized to 16S cDNA (diluted 1:10,000) and the calculation of the relative gene expression level was done according to the 2^-ΔΔCT^ method (Schmittgen and Livak, 2008), except where stated otherwise. For statistical analysis, student’s *t*-test was performed on the ^ΔCT^-values; *p*-values lower than 0.05 were regarded as significant.

## Results

### Transcript Levels of Hydrogenase Genes under Different Growth Conditions

To determine and compare the transcript levels of the hydrogenase genes under different growth conditions, *S. multivorans* was grown with the following substrate combinations: pyruvate plus fumarate (standard condition), PCE, 5% O_2_, or without external electron acceptor, or H_2_ plus PCE or nitrate. In addition, N_2_-fixing conditions were achieved with N_2_ as sole nitrogen source. Prior to the main qPCR experiments, three housekeeping genes were tested for their suitability as reference genes: 16S rRNA, *recA* and *rpoB*. The latter was not considered further due to unspecific products and primer specificities out of an acceptable range (more than 10% deviation from primer specificities for the other primer pairs). The *recA* gene was unsuitable, since the transcript levels varied too much among replicates and under the different growth conditions (see Supplementary Data Sheet 2). Therefore, hydrogenase qPCR data were normalized to the 16S rRNA gene when comparing transcript levels under different growth conditions. For hydrogenase qPCR, the following genes encoding the catalytic subunits of each hydrogenase gene cluster were chosen: *hydB* (SMUL_1424) of the MBH gene cluster, *hupL* (SMUL_1422) from the cytoplasmic uptake hydrogenase (*hupSL*), *echE* (SMUL_1307) from the CooH-like hydrogenase and *hyfG* (SMUL_2388) from the Hyf hydrogenase. Primers used in qPCR are listed in Supplementary Table 1.

**FIGURE 2:**
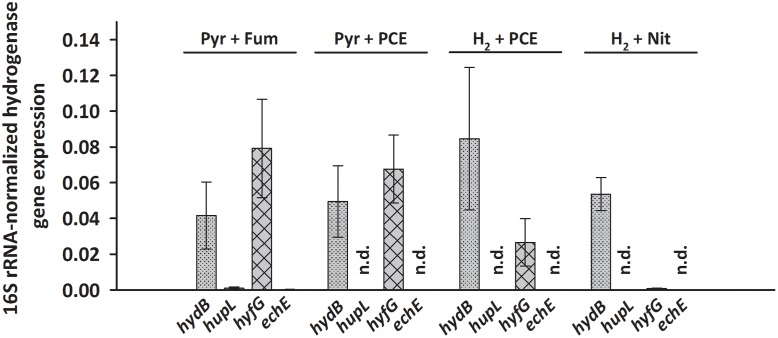
**Transcription pattern of hydrogenase catalytic subunit genes of *S. multivorans* grown under different growth conditions.** Transcript levels of hydrogenase genes were normalized to those of the 16S rRNA gene. All data were obtained from three biological replicates and three technical replicates. When amplification was detected only in one biological replicate, the hydrogenase gene was designated as not detected (n.d.). The raw data of all values are shown in the Supplementary Data Sheet 2. *hydB*, membrane-bound hydrogenase (MBH); *hupL*, cytoplasmic uptake hydrogenase; *hyfG*, Hyf-hydrogenase; *echE*, Ech-like hydrogenase. Pyr, pyruvate; Fum, fumarate; Nit, nitrate; PCE, tetrachloroethene.

Transcripts of only two hydrogenase genes, *hydB* and *hyfG* (large subunits of the MBH and of Hyf) were found at levels of 0.03 to 0.08 (after normalization to 16S rRNA transcript level) under nearly all tested growth conditions. This was even the case when pyruvate rather than H_2_ was used as electron donor in cells cultivated for at least 50 transfers with pyruvate as sole energy source. A long-term transcriptional regulatory effect, as was seen for *pceA* (John et al., 2009), can therefore be excluded. Opposed to *hydB* and *hyfG*, *hupL* and *echE* transcripts were not detected under most conditions, except very low transcript levels under pyruvate/fumarate (below 0.001, see **Figure 2** and Supplementary Data Sheet 2). To establish the hydrogenases that are involved in H_2_-dependent PCE respiration, *S. multivorans* was grown in the presence of 100% H_2_ in the gas phase as electron donor and 10 mM PCE in a hexadecane phase as electron acceptor. Only an insignificant increase in *hydB* transcript level (about twofold, *p*-value 0.224) and a decrease of *hyfG* (about threefold, *p*-value 0.562) was observed (**Figure 2**). To compare these transcript levels to those of another anaerobic H_2_-dependent respiration, cells were grown with nitrate as electron acceptor and H_2_ as electron donor. Nitrate rather than fumarate was chosen as electron acceptor, since *S. multivorans* was shown to be capable of growth with fumarate alone by disproportionation (Scholz-Muramatsu et al., 1995). The *hydB* gene transcript level dropped slightly, but insignificantly compared to H_2_/PCE (twofold, *p*-value 0.384), while *hyfG* was reduced significantly in H_2_/nitrate cells (about 30-fold lower; *p*-value 0.022) (**Figure 2**).

**FIGURE 3:**
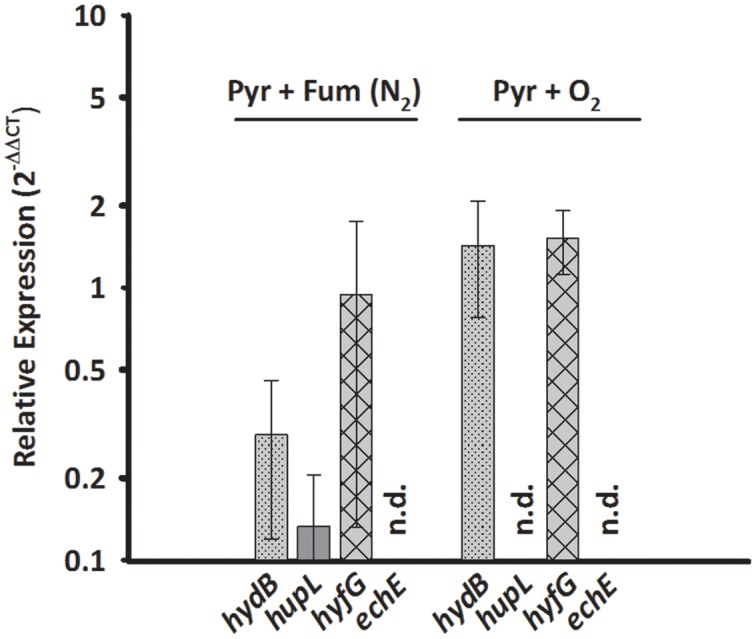
**Transcription pattern of hydrogenase catalytic subunit genes of *S. multivorans* with N_2_ as sole N-source and 5% O_2_ as electron acceptor.** Transcript levels obtained from cells grown with N_2_ as N-source and 5% O_2_ were normalized to the transcript levels of the corresponding hydrogenase genes of pyruvate/fumarate grown cells. All data were obtained from three biological replicates and three technical replicates. When amplification was detected only in one biological replicate, the hydrogenase gene was designated as not detected (n.d.). The raw data of all values are shown in the Supplementary Data Sheet 2 and Figure 1 shows the data normalized to 16S rRNA gene. *hydB*, membrane-bound hydrogenase (MBH); *hupL*, cytoplasmic uptake hydrogenase; *hyfG*, Hyf-hydrogenase; *echE*, Ech-like hydrogenase. Pyr, pyruvate; Fum, fumarate; NH_4_Cl, with ammonium chloride; N_2_, N_2_ as sole N-source.

Since HupSL might be involved in recycling hydrogen produced during N_2_ fixation, as was shown for similar enzymes in cyanobacteria (Tamagnini et al., 2007; Bothe et al., 2010) and *S. multivorans* was shown to fix N_2_ (Ju et al., 2007; Goris et al., 2014), the transcript levels of *hupL* were investigated with N_2_ as sole N-source. *S. multivorans* was grown under standard conditions (pyruvate/fumarate plus NH_4_Cl as N-source) and with medium containing N_2_ as sole N-source. The *hupL* transcript level did not increase with N_2_ as sole N-source when compared to cells grown with NH_4_^+^. Instead, *hupL* and *hydB* levels decreased about 7.5- and 3-fold, albeit insignificantly with a *p*-value of 0.592 and 0.892, respectively (**Figure 3**).

Aerobic growth of *S. multivorans* with pyruvate and an O_2_-concentration of about 5% in the gas phase was described recently (Goris et al., 2014). Since NiFe hydrogenases are generally inactivated by O_2_ (Stiebritz and Reiher, 2012), it was of interest whether O_2_ could possibly cause a down-regulation of hydrogenase gene expression as found for other bacteria (Kovács et al., 2005). Therefore, qPCR of cells grown in the presence of 5% O_2_ in the gas phase with pyruvate as electron donor was performed to test if hydrogenase transcription occurs in the presence of oxygen. Cells were harvested at an O_2_ concentration in the medium of at least 0.2 mg/L. Surprisingly, the transcript levels of *hydB* and *hyfG* were similar or insignificantly higher (about twofold for *hydB*, *p*-value of 0.553) under microoxic conditions compared to cells grown with fumarate as electron acceptor (**Figure 3**), while *hupL* and *echE* transcripts were not detected. The cells were, however, not able to grow with H_2_ as electron donor and 5% O_2_ as electron acceptor and acetate as carbon source.

**FIGURE 4:**
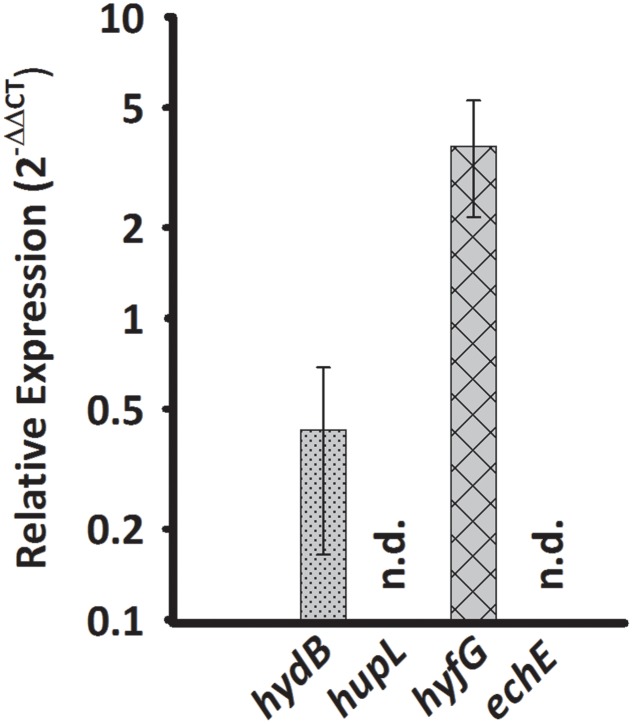
**Transcription pattern of hydrogenase catalytic subunit genes of *S. multivorans* grown fermentatively with pyruvate.** Transcript levels obtained from cells grown with pyruvate in the absence of an electron acceptor were normalized to the transcript levels of the corresponding hydrogenase genes of pyruvate/fumarate grown cells. All data were obtained from three biological replicates and three technical replicates. When amplification was detected in less than two biological replicates, the hydrogenase gene was designated as not detected (n.d.). The raw data of all values are shown in the Supplementary Data Sheet 2 and Figure 1 shows the data normalized to 16S rRNA gene. *hydB*, membrane-bound hydrogenase (MBH); *hupL*, cytoplasmic uptake hydrogenase; *hyfG*, Hyf-hydrogenase; *echE*, Ech-like hydrogenase. Pyr, pyruvate; Fum, fumarate.

*Sulfurospirillum multivorans* was described to grow fermentatively with pyruvate as the sole energy source (Scholz-Muramatsu et al., 1995). To investigate whether one of the putative H_2_-producing hydrogenases shows higher transcript levels under this growth condition, we compared qPCR results obtained from pyruvate-grown cells with those from pyruvate/fumarate-grown cells. The *hydB* transcript level was lowered insignificantly (*p*-value 0.229) about threefold, while the *hyfG* level was increased by a factor of 3 with a *p*-value of 0.056 (**Figure 4**).

### Biochemical Characterization of the Membrane-Bound Hydrogenase

**FIGURE 5:**
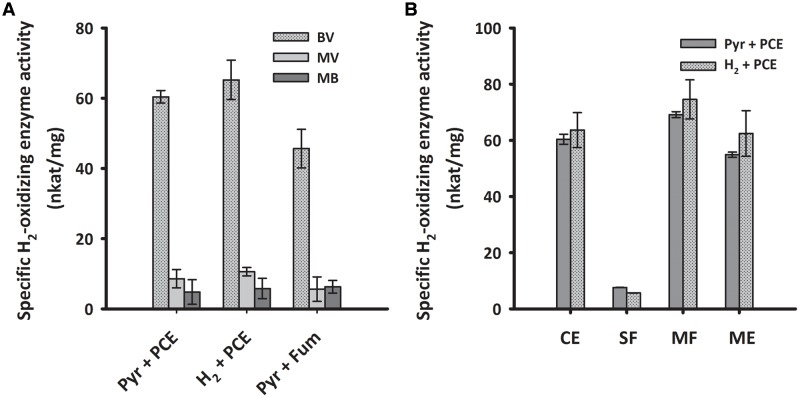
**H_2_-oxidizing activities of crude extracts with different redox dyes** **(A)** and enzyme activity of subcellular fractions of *S. multivorans* measured with benzyl viologen **(B)**. **(A)** Specific H_2_-oxidizing enzyme activity (nkat/mg) of crude extracts from cells grown under different cultivation conditions. **(B)** Specific H_2_-oxidizing enzyme activity (nkat/mg) of different cell fractions from pyruvate/PCE and H_2_/PCE-grown cells. Data are calculated from three independent biological and technical replicates. Cells were fractionated according to the “Experimental Procedures” section and solubilization was done with 0.1% Triton X-100. For concentrations of redox dyes, see section “Experimental Procedures”. BV, benzyl viologen; MV, methyl viologen; MB, methylene blue. Pyr, pyruvate; Fum, fumarate; CE, crude extract; SF, soluble fraction; MF, membrane fraction; ME, membrane extract.

To biochemically characterize the H_2_-oxidizing enzyme of cells grown under PCE-respiring conditions, we measured the H_2_-uptake activity of subcellular fractions from *S. multivorans* in different enzymatic assays. First, crude extracts of cells grown with pyruvate or H_2_ as electron donor and PCE as electron acceptor were tested spectrophotometrically for H_2_-oxidizing activity with several different artificial redox mediators as electron acceptors. The activity with BV was by far the highest and about five times higher than the activities with MV and approximately tenfold of the activity with MB (**Figure 5A**). The other redox mediators tested, NAD^+^, nitro blue tetrazolium chloride (NBT) or phenazine methosulfate (PMS), and negative controls without H_2_ or without cell extracts showed no activity (Supplementary Table 2). In accordance with the quantitative PCR results, there was not much difference in the activity levels between pyruvate/PCE- and H_2_/PCE-grown cells, with a specific H_2_-oxidizing activity of 60 nkat/mg protein with BV as electron acceptor for pyruvate/PCE cells, 50 nkat/mg for pyruvate/fumarate cells and 70 nkat/mg for H_2_/PCE cells (**Figure 5A**). Crude extracts obtained from cells grown with pyruvate as electron donor and 5% O_2_ as electron acceptor showed H_2_-dependent enzyme activity with BV (25 ± 2.3 nkat/mg). After subcellular fractionation, the highest specific activity (about 60 nkat/mg) was found in the MF while only around one tenth (7 and 5 nkat/mg for extracts obtained from pyruvate- and H_2_-grown cells, respectively) was measured for the SF. The activities of crude extract, MF and solubilized MEs were about 50 to 70 nkat/mg (**Figure 5B**).

**FIGURE 6:**
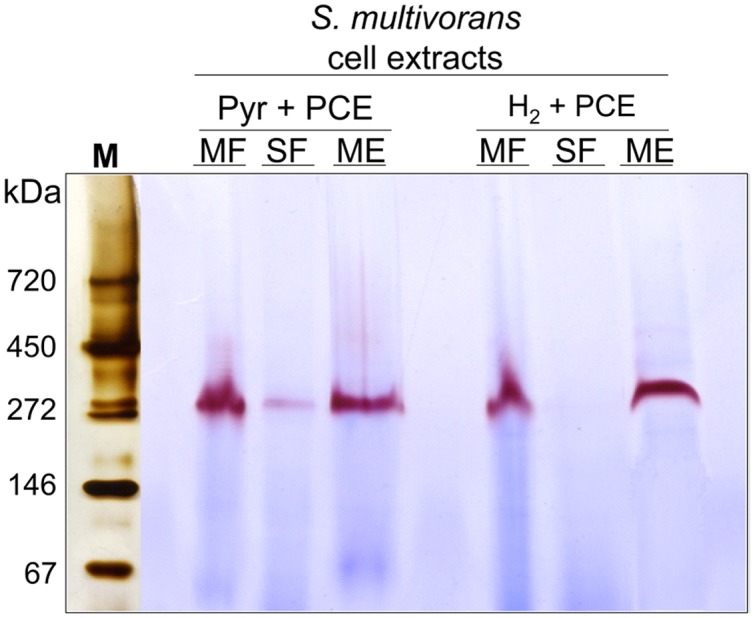
**Analysis of the localization and oligomeric organization of the H_2_-oxidizing enzyme of *S. multivorans*.** Blue native activity stained gels of *S. multivorans* cell fractions are shown. Marker lane was cut off and silver stained separately. One microgram protein was applied to each lane. Cells were grown with pyruvate/PCE or H_2_/PCE. MF, membrane fraction; SF, soluble fraction; ME, membrane extract; M, marker lane.

The different subcellular fractions were subjected to native PAGE followed by activity staining. This method allows to estimate the size and activity of catalytically active enzyme complexes and is often routinely used for the characterization of hydrogenases (Ballantine and Boxer, 1985; Pinske et al., 2012). For this purpose, *S. multivorans* cells were grown with either pyruvate or H_2_ as electron donor and PCE as electron acceptor. Both, cell extracts and subcellular fractions (SF and ME solubilized with digitonin), were subjected to activity staining with BV as primary and TTC (triphenyl tetrazolium chloride) as secondary electron acceptor. After incubation of the gel with H_2_ for 15–30 min at 28°C, a distinct band showing the typical reddish color of reduced TTC appeared at a size of about 270 kDa. This band was missing or only weakly visible in the SF (**Figure 6**). A prolonged incubation time up to 12 h did not lead to an additional band in the activity staining with membrane or SFs, suggesting the involvement of only one hydrogenase in H_2_ oxidation, albeit a hydrogenase not performing H_2_ oxidation under the given conditions might have been overlooked in this assay. The size of 270 kDa correlates to a dimeric form of the heterotrimeric *S. multivorans* MBH, which is predicted to be around 244 kDa (predicted sizes for the maturated MBH – large subunit HydB: 62 kDa, small subunit HydA: 34 kDa, and the membrane-integral cytochrome *b*, HydC: 26 kDa). This value, however, does not take into account the unknown contribution of the digitonin micelle size.

The presumable physiological electron acceptor for the MBH is menaquinone, as was shown for the MBH from *W. succinogenes* (Dross et al., 1992). To test the H_2_-dependent reactivity of the MBH of *S. multivorans* with quinones, H_2_ oxidation activity was measured spectrophotometrically with two different quinone analogs. Similar to the MBH of *W. succinogenes*, crude and MEs of *S. multivorans* showed activity with 2,3-dimethyl-1,4-naphtoquinone (DMN) (**Table 1**). Extracts from cells grown with H_2_ showed approximately 10% higher activities than those from cells grown with pyruvate (**Table 1**). The hydrophilic quinone analog, 1,4-naphthoquinone, was neither reduced by the MBH of *S. multivorans* nor by that of *W. succinogenes* (Dross et al., 1992).

**Table 1:** H_2_-oxidizing activities of different cell fractions with DMN as electron acceptor.

Growth substrates	Cell fraction	Specific hydrogenase activity (nkat/mg)
		H_2_ –> DMN
Pyruvate + PCE	ME	41.9 ± 4.67
	MF	26.9 ± 3.14
H_2_ + PCE	ME	54.4 ± 5.57
	MF	29.9 ± 6.38


*DMN, 2,3-dimethyl-1,4-naphthoquinone; MF, membrane fraction; ME, membrane extract (solubilized membranes).*

**FIGURE 7:**
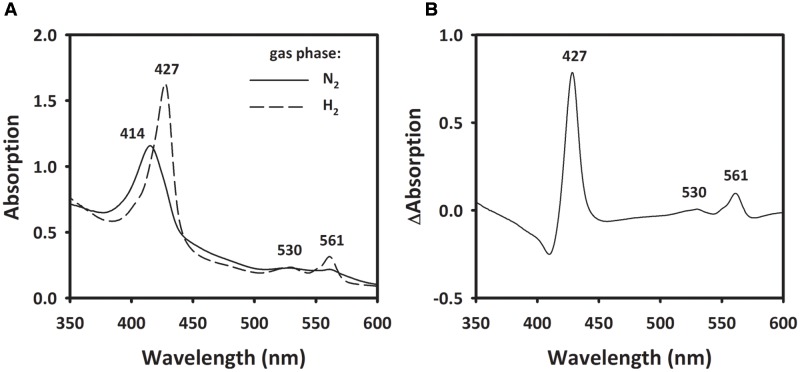
**Absorption spectra of membrane extract of *S. multivorans*.** **(A)** The solid line represents the membrane fraction under N_2_ as gas phase. Incubation of the sample for 10 min under H_2_ leads to the spectrum represented by the dashed line **(B)** Difference spectra of the H_2_-reduced minus N_2_-incubated state. Numbers indicate wavelengths of characteristic absorption peaks of *b*-type cytochromes. Cells were grown with pyruvate/PCE.

To investigate the involvement of a cytochrome *b*, spectroscopic changes in UV/VIS absorption of MFs were recorded before and after the addition of H_2_. The MF showed an absorption maximum at 414 nm in the absence of H_2_. After flushing the sample with H_2_, the Soret band at 414 nm shifted to 427 nm and two peaks at 530 and 561 nm appeared (**Figure 7A**). When calculating the difference spectra of H_2_-reduced minus oxidized state, the spectrum showed α-, β-, and γ-absorption peaks at 561, 530, and 427 nm, respectively (**Figure 7B**). These spectral properties correspond to absorption spectra of *b*-type cytochromes (Yoon et al., 2008; Eguchi et al., 2012).

### Enrichment of the Membrane-Bound Hydrogenase

**FIGURE 8:**
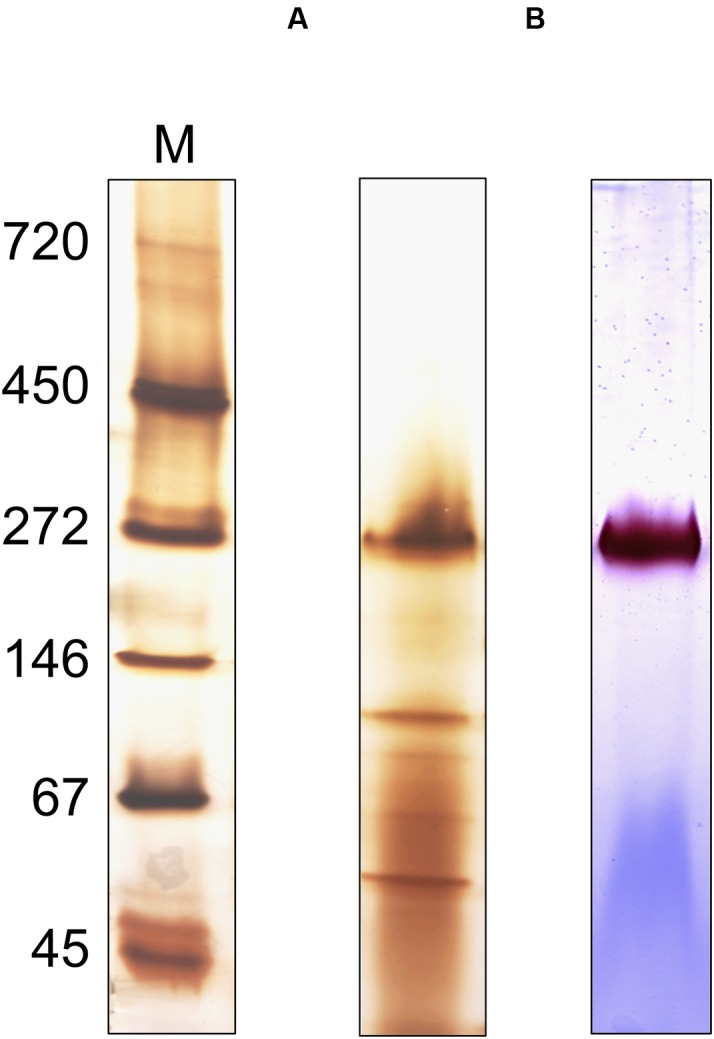
**Oligomeric analysis of the enriched MBH of *S. multivorans*.** **(A)** Silver-stained Native PAGE and **(B)** activity stained BN-PAGE of the enriched MBH. Ten microgram of protein was applied on native PAGE and 1 μg for hydrogenase activity stain. M: marker lane. Cells were grown with pyruvate/fumarate.

Attempts to express the MBH of *S. multivorans* heterologously did not lead to the formation of an active enzyme, therefore, an enrichment from MEs was carried out (**Figure 8**). After solubilization of the MF with digitonin, 18% of hydrogenase enzyme activity from the MF could be recovered. An enrichment of 221-fold was obtained using anion exchange chromatography with a Q-Sepharose column (**Table 2**). After elution, the fractions with the highest activities (fractions 19–23) were applied to SDS-PAGE, native PAGE and BN activity staining (Supplementary Figure 2). In the latter, all fractions showed the remarkable red band at 270 kDa, which was most intense in fractions 20 and 21. Similar to the cell extracts, no second band was visible after prolonged incubation (up to 12 h) in the reaction mixture. Silver stained native PAGE showed the same 270 kDa band in all applied fractions with the most intense band in fraction 20 (**Figure 8A**). Other distinct bands in fraction 20 appeared around 50 and 130 kDa. After SDS-PAGE, all tested fractions showed a band at about 65 kDa, which corresponds to the size of HydB (62 kDa). This band was strongest in fractions 20 and 21. Additionally, a band at nearly 35 kDa was showing up in fractions 20 to 23, correlating to the theoretical size of the mature small subunit of the MBH (34 kDa). Several bands between 25 and 30 kDa were visible in all fractions, but none of them could be attributed clearly to HydC (26 kDa). One predominant band at 25 kDa observed in fractions 20 and 21 might represent a cytochrome *b*, especially since both fractions showed activity with DMN as electron acceptor (Supplementary Figure 2). Fraction 20 appeared to exhibit less additional bands than fraction 21 after SDS-PAGE (Supplementary Figure 2), therefore, we chose this fraction for further purification. Hydrophobic interaction chromatography or gel filtration did not lead to further enrichment of active trimeric MBH, so that further characterization was performed with fraction 20. The presence of the membrane-integral cytochrome *b* (HydC) in this fraction was shown using UV/Vis spectroscopy. The same characteristic absorption spectra and difference spectrum were recorded as those obtained for the MF (Supplementary Figure 3). Compared to the membranes, the enriched MBH showed a higher absorption in the difference spectrum, suggesting a higher cytochrome *b* concentration. The optimal condition for H_2_ oxidation by fraction 20 was at pH 8.0 and 40°C when BV was used as electron acceptor in the photometric assay.

**Table 2:** Enrichment of membrane-bound hydrogenase of *S. multivorans*.

Purification step	Activity (nkat/ml)	Protein (mg/ml)	Specific activity (nkat/mg)	Yield (%)	Purification (fold)
Membrane fraction	92	20	4.6	100	1
Digitonin solubilization	403.1	3.6	112	18	24.3
Q-Sepharose HP	1326	1.3	1020	6.5	221


*Enzyme activity was measured with benzyl viologen. Cells were grown with pyruvate/fumarate.*

## Discussion

To identify hydrogenases involved in the hydrogen-oxidation during PCE respiration in *S. multivorans*, we performed transcription analysis and biochemical investigations with cells grown in the presence of different electron donor/acceptor combinations. Of the previously identified four NiFe hydrogenases encoded in the genome of *S. multivorans*, a membrane-bound hydrogenase similar to the hydrogen-oxidizing MBH from *W. succinogenes* was suggested to play a role in hydrogen oxidation during PCE respiration. However, the only evidence for this assumption was based on amino acid sequence similarities to other hydrogen-oxidizing enzymes and cell extracts grown with H_2_ and fumarate (Miller et al., 1996; Goris et al., 2014), which did not allow for an unambiguous conclusion of its role in PCE respiration. Since up to now no detailed information on the composition of organohalide respiratory chains is available, it is feasible that one of the other three hydrogenases might be involved. In the transcript level analysis presented here, only two of the four hydrogenase large subunit genes were found to be transcribed to a considerable amount. The first is *hydB*, encoding the large subunit of the MBH similar to that of *W. succinogenes*, while the second, *hyfG*, belongs to a group 4 hydrogenase (Vignais and Billoud, 2007) similar to hydrogenases 3 and 4 of *E. coli* (Hyc and Hyf). The *hydB* transcript level was found not to be significantly altered during any of the tested growth conditions. The presence of the MBH and Hyf with pyruvate or formate as electron donor was already seen in a previous proteomic study (Goris et al., 2015) (Supplementary Data Sheet 3). This points toward a constitutive expression of the MBH in *S. multivorans*. As this was tested in cells long-term cultivated without H_2_, a regulatory effect as reported earlier for PCE respiration (John et al., 2009) can be also excluded.

**FIGURE 9:**
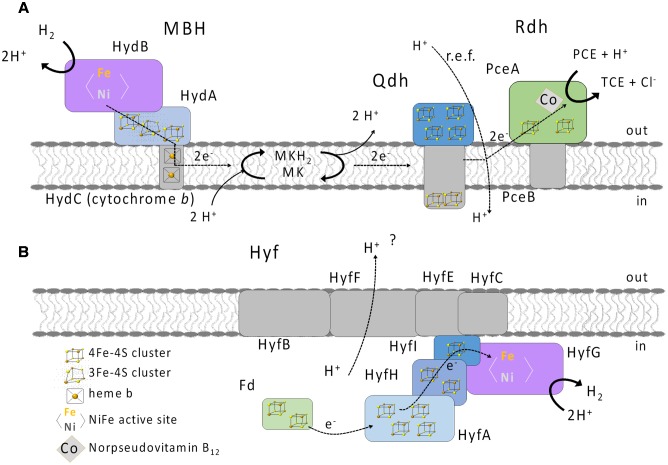
**Putative physiological roles of the MBH** **(A)** and Hyf hydrogenase **(B)** in *S. multivorans*. Qdh, quinol dehydrogenase; PceAB, PCE reductive dehalogenase; MK, menaquinone; MKH2, reduced MK; Fd, ferredoxin; PCE, tetrachloroethene; TCE, trichloroethene; i, inside (cytoplasm); o, outside (periplasm).

Post-transcriptional regulation and maturation could also have an influence on the hydrogen-oxidizing activity and the responsible hydrogenase. The biochemical analyses with extracts obtained from cells grown under different conditions revealed comparable H_2_-oxidizing activities regardless of the electron donor or acceptor used. Also, H_2_ had no apparent influence on the maturation of the MBH, since the H_2_-oxidizing activity in the ME did not change when cultures were grown with H_2_. Furthermore, we conclude that only one hydrogenase plays a role in hydrogen oxidation under all growth conditions tested, since only one band was detected in BN activity staining regardless of the growth condition. However, the blue native activity staining was performed only with the primary electron acceptor BV. Therefore, the presence of another hydrogen-oxidizing enzyme requiring a different electron acceptor cannot be completely ruled out. Activity-stained BN PAGE of a hydrogen-oxidizing enzyme enriched from *S. multivorans* showed a band corresponding to the same size. SDS PAGE analysis of the subunit composition revealed subunits with molecular masses corresponding to the three subunits HydABC of the MBH. Spectroscopic analysis of the enriched enzyme revealed characteristic spectra of *b*-type cytochromes, indicating the presence of a cytochrome *b* in the enrichment. These results are in accordance with spectra obtained from membranes and from the purified MBH of *W. succinogenes*. In addition, the H_2_-dependent reduction of the quinone analog DMN indicates that electrons derived from H_2_ oxidation are transferred via the membrane-integral cytochrome *b* to the menaquinone pool (**Figure 9A**). Previous studies suggested the involvement of menaquinone and a quinol dehydrogenase in the PCE respiratory chain (Goris et al., 2014, 2015), which supports the assumption that the MBH-mediated hydrogen oxidation is coupled to PCE reduction via menaquinone.

The apparent molecular mass of the native enzyme points to a heterotrimeric MBH dimer. This is opposed to a putative trimeric composition of the heterotrimeric complex of *Cupriavidus necator* MBH (Frielingsdorf et al., 2011). Instead, the *S. multivorans* complex resembles the *E. coli* hydrogenase 1 crystallized as a heterotrimer (Volbeda et al., 2013), albeit the latter was reported to contain only one cytochrome for a hydrogenase dimer. A dimeric structure is not only found for *E. coli* hydrogenase 1, but also found in several other hydrogenase crystal structures, although these were solved without a membrane-integral subunit (Wulff et al., 2016).

The broad array of growth conditions used for real-time PCR experiments in this study revealed that under none of the conditions tested neither *hupL* nor *echE* were induced. In contrast, *hydB* was expressed under all growth conditions, even with O_2_ as electron acceptor. The latter observation was surprising, since Epsilonproteobacteria including *S. multivorans* do not harbor oxygen-tolerant NiFe hydrogenases and most NiFe hydrogenases suffer from inactivation when exposed to O_2_. Therefore, many facultative anaerobic bacteria employ mechanisms to down-regulate the expression of hydrogenase genes in the presence of O_2_. For example, hydrogenase gene transcription is under control of regulators ArcAB and Fnr in other facultative anaerobic proteobacteria including *E. coli* (Richard et al., 1999; Kovács et al., 2005). None of these regulators is found in Epsilonproteobacteria, which might explain the lack of O_2_-induced down-regulation of hydrogenase gene expression. Many free-living Epsilonproteobacteria are facultative microaerophilic and often found in the oxic–anoxic interface, where the O_2_ concentration varies (Vetriani et al., 2003; Grote et al., 2012). Therefore, a constitutive expression of the hydrogen-uptake system and the lack of an O_2_-sensitive regulation system may be beneficial to these organisms. Although all attempts to grow *S. multivorans* with H_2_ and O_2_ as energy substrates failed so far, it is feasible that the organism might use H_2_ as additional electron donor under very low O_2_ partial pressure as described elsewhere for other Epsilonproteobacteria (e.g., *Campylobacter jejuni* and *Helicobacter pylori*) (Laanbroek et al., 1977, 1978; Carlone and Lascelles, 1982; Maier et al., 2003). Since crude extracts from cells grown with O_2_ still showed around half of the activity as anaerobically grown cells, this seems feasible for *S. multivorans*.

The presence of H_2_ in the gas phase did not raise the transcript level of the MBH significantly. On the molecular level, this might be attributed to a missing H_2_ sensor such as that found in some “Knallgas” bacteria (Kleihues et al., 2000; Friedrich et al., 2005). The absence of regulatory proteins with the exception of the nickel-dependent NikR (Goris et al., 2014) is in accordance with a constitutive expression of the MBH in *S. multivorans*. The constitutive expression of the MBH might also be an advantage in anoxic environments where nutrient composition and H_2_ levels are often changing.

The role of the Hyf hydrogenase in *S. multivorans* remains enigmatic. The key enzyme in mixed acid fermentation in *E. coli* is the pyruvate formate lyase (PFL), generating formate, which is the substrate for the FHL complex. An ortholog of the PFL is not encoded in the genome of *S. multivorans*. While the amino acid sequences of *S. multivorans* Hyf subunits are similar (33 to 50% amino acid sequence identity) to those of hydrogenase 4 of *E. coli*, the *S. multivorans hyf* gene cluster does not contain any genes encoding formate-metabolism related proteins as found in the *hyf* cluster of *E. coli* (e.g., the formate channel *focB* or a formate-sensitive transcriptional regulator). A gene encoding a putative cytoplasmic formate dehydrogenase is found in the genome of *S. multivorans*, but the according protein sequence shows only 30% amino acid sequence identity to FdhF of the FHL complex of *E. coli*. Additionally, an ortholog of the *S. multivorans* Fdh is found in many epsilonproteobacterial genomes not encoding the Hyf hydrogenase. All this renders participation of Hyf in an FHL complex in *S. multivorans* an unlikely scenario. Instead, we assume that Hyf could serve to dispose of excess reducing equivalents, accepting electrons, e.g., from ferredoxin reduced by a pyruvate:ferredoxin oxidoreductase (PFOR), similar to the group 4 hydrogenase of *Pyrococcus furiosus* (McTernan et al., 2015). The ferredoxin and the PFOR were found to be present in the proteome in *S. multivorans* previously (Goris et al., 2015). We assume that *S. multivorans* produces H_2_ especially under fermentative conditions (**Figure 9B**) or when the concentration of the electron acceptor may be limiting. This is probably the case for PCE, which, even in polluted environments, could be present at low concentrations due to its low solubility. In contrast, nitrate may be available at higher concentrations, which might explain the down-regulation exclusively with H_2_/nitrate as substrates. The role and function of this hydrogenase is subject to further studies currently conducted in our laboratory.

The role of the cytoplasmic uptake hydrogenase HupSL remains unclear in *S. multivorans*. A TetR-like transcriptional repressor which is encoded directly upstream of the *hupSL* cluster (Goris et al., 2014) might repress the transcription of the enzyme under the tested growth conditions. The function of HupSL in recycling of H_2_ as a byproduct of N_2_ fixation can be excluded, since growth with N_2_ as sole N-source did not lead to an elevated transcript level, which would have been expected in that case. A role in reducing a cytoplasmic low-potential electron donor such as reduced ferredoxin providing electrons for the reductive TCA cycle seems feasible, as was suggested for a similar hydrogenase in *A. aeolicus* (Guiral et al., 2005). The proteins for the rTCA cycle are encoded in the genome of *S. multivorans* (Goris et al., 2014), but autotrophic growth was never observed. Like for the *hupL* gene, also *echE* transcripts were only found in negligible amounts in this study. All Ech subunits bear high similarity to the CO-induced hydrogenase of *C. hydrogenoformans*, in which the hydrogenase genes are located adjacent to a CO dehydrogenase gene (Soboh et al., 2002). In the genome of *Sulfurospirillum* sp. SCADC, this structure is still conserved (Tan and Foght, 2014; Goris and Diekert, 2016) and *S. carboxydovorans* is reported to use CO as electron donor (Jensen and Finster, 2005). Hence, a loss of the CO dehydrogenase gene and non-functionality of the CO-induced like hydrogenase in *S. multivorans* is possible. However, it is also feasible that growth conditions, which induce transcription of the *hup* and *ech* genes, were not tested in this study. For example, pH variation or different levels of H_2_ concentrations, which could have an effect on hydrogenase expression, was not included here.

## Conclusion

In this study we showed that *S. multivorans* involves a membrane-bound NiFe hydrogenase similar to the MBH of *W. succinogenes* for H_2_ oxidation in the PCE-respiratory chain. The MBH, which was enriched with a redox-active, membrane-integral cytochrome *b*, reacts most likely with the quinone pool and forms a native complex of around 270 kDa. The transcript studies presented here show that the MBH is not subject to a distinct H_2_- or O_2_-dependent regulation as reported for other organisms. Besides the MBH, only one more hydrogenase gene transcript (Hyf type) was found under the growth conditions applied in this study. This cytoplasmic, membrane-bound hydrogenase complex with similarities to Hyf from *E. coli* is most likely playing a role in proton reduction under electron acceptor-limiting conditions.

Taken together, this study allows a deeper insight into the H_2_ metabolism of a model organohalide-respiring bacterium and a free-living Epsilonproteobacterium.

## Author Contributions

TG and GD initiated and supervised the study, SK and TG designed experiments, analyzed data and drafted the manuscript. SK performed the biochemical and real-time PCR experiments, MW performed initial real-time PCR experiments, including primer design and testing of optimal conditions, XW performed initial biochemical experiments. All authors curated, read, and approved the final manuscript.

## Conflict of Interest Statement

The authors declare that the research was conducted in the absence of any commercial or financial relationships that could be construed as a potential conflict of interest.
