# Supplementary material for: The NiFe Hydrogenases of the Tetrachloroethene-Respiring Epsilonproteobacterium Sulfurospirillum multivorans: Biochemical Studies and Transcription Analysis
Source: Front Microbiol. 2017 Mar 20;8:444. doi: 10.3389/fmicb.2017.00444 (PMC5357620; doi:10.3389/fmicb.2017.00444)
Supplement: Supplementary file 1 [file Data_Sheet_1.docx]

Supplementary Material

The NiFe Hydrogenases of the Tetrachloroethene-Respiring Epsilonproteobacterium *Sulfurospirillum multivorans*: Biochemical and Transcription Studies

**Stefan Kruse^1*^, Tobias Goris^1*^, Maria Wolf^1,2^, Xi Wei^1,3,4^, Gabriele Diekert^1^**

^1^Department of Applied and Ecological Microbiology, Institute of Microbiology, Friedrich Schiller University, 07743 Jena, Germany

^2^Dianovis GmbH, Wichmannstr. 12, 07973 Greiz

^3^Department Isotope Biogeochemistry, Helmholtz Centre for Environmental Research – UFZ, Permoserstr. 15, 04318 Leipzig, Germany

^4^YMC Europe GmbH, Schöttmannshof 19, Dinslaken, 46539, Germany

*These authors contributed equally

*** Correspondence:**

Tobias Goris
tobias.goris@uni-jena.de

**Supplementary Figure 1: Transcription pattern of hydrogenase catalytic subunit genes of *S. multivorans* with N_2_ as sole N-source (A) and O_2_ (B) as electron acceptor and under pyruvate fermentation (C).** Transcript levels are normalized to the *16S* rRNA gene. All data were obtained from three biological replicates and three technical replicates. When amplification was detected only in one biological replicate, the hydrogenase gene was designated as not detected (n.d.). *hydB* - membrane-bound hydrogenase (MBH), *hupL* - cytoplasmic uptake hydrogenase, *hyfG* - Hyf-hydrogenase, *echE* - Ech-like hydrogenase. Pyr - pyruvate; Fum - fumarate; NH_4_Cl - with ammonium chloride; N_2_ - N_2_ as sole N-source.

**
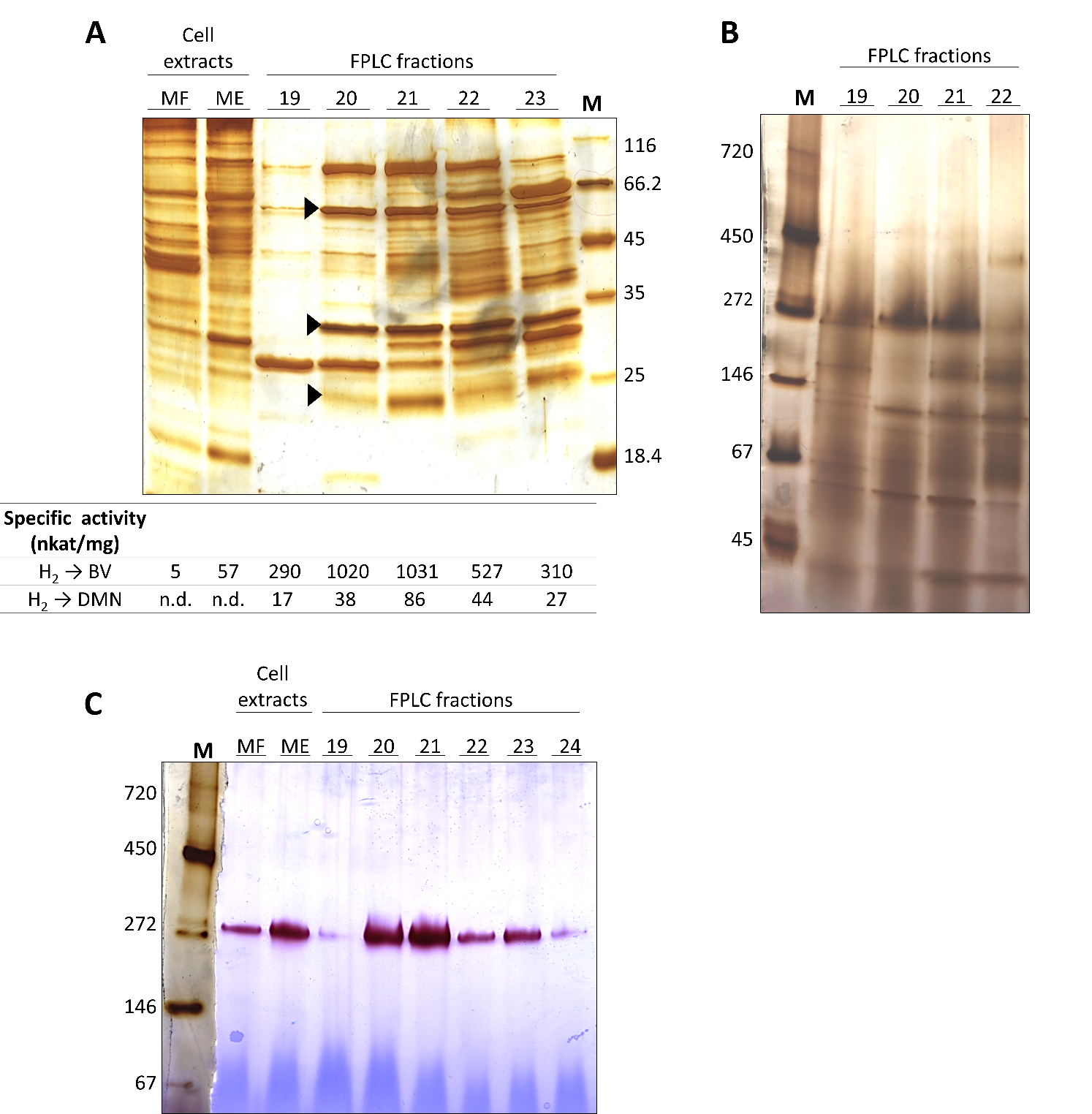
**

**Supplementary Figure 2: Analysis of fractions obtained after FPLC purification according enrichment and oligomeric organization of the hydrogen oxidizing enzyme of *S. multivorans*.** (A) Silver stained SDS polyacrylamide gel electrophoresis of cell extracts and FPLC fractions. 10 µg of protein was applied. Specific activities of fractions with BV and DMN are depicted in the table below. Arrows are indicating sizes/bands of the predicted MBH subunits, from top to bottom: HydB, HydA, HydC. HydB and HydA could be detected via MS in a different purification not shown here. (B) Non denaturing PAGE of FPLC fractions. 5 µg protein was applied. (C) Hydrogenase activity stained Blue Native PAGE of cell extracts and FPLC fractions. Marker band was cut off and silver stained. 1 µg of protein was applied. MF: membrane fraction, ME: membrane extract, M: marker lane, BV: benzyl viologen, DMN: 2,3-dimethyl-1,4-naphthoquinone, n.d.: not determined.

**
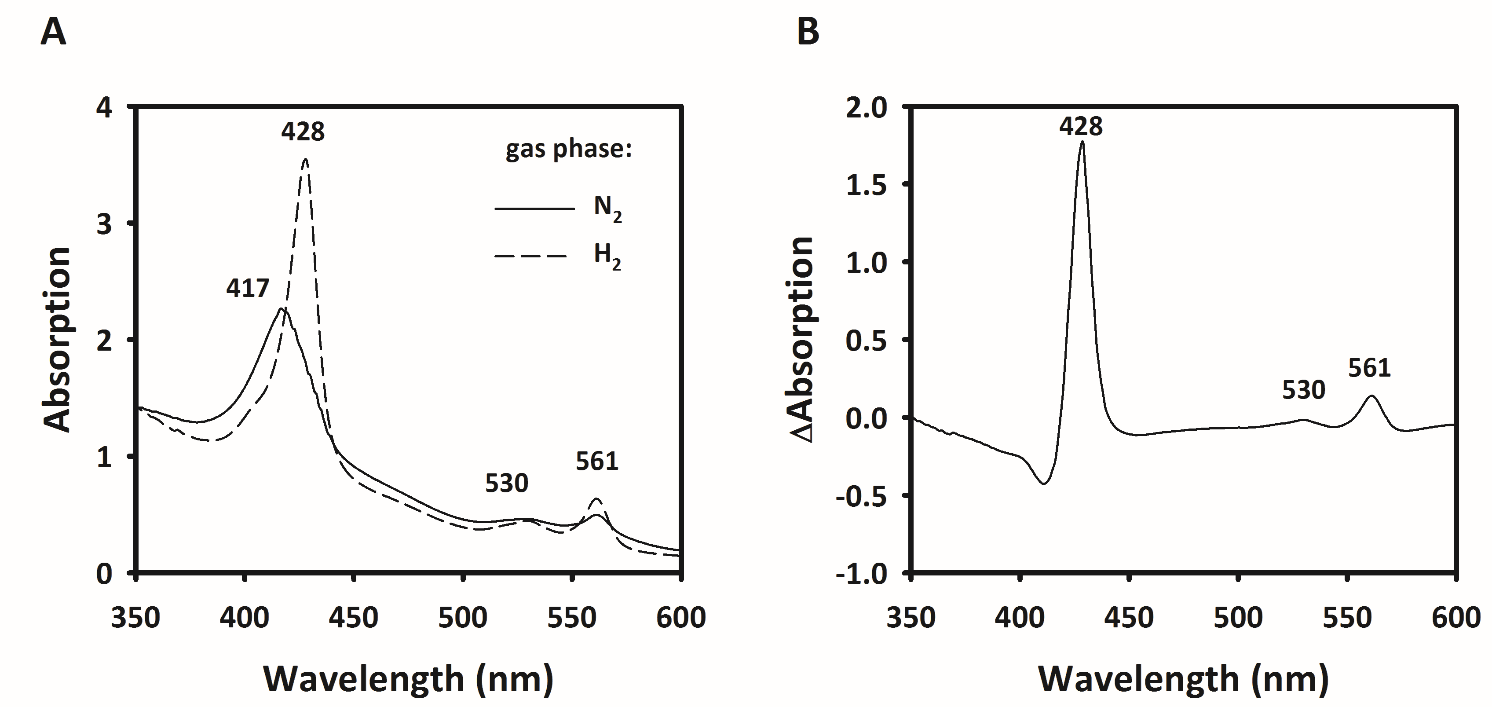
**

**Supplementary Figure 3: Absorption spectra (A) and difference spectra (B) of the enriched MBH of *S. multivorans*.** Cells were grown with pyruvate and fumarate. Spectra were obtained from fraction 20.

**Supplementary Table 1: Bacterial strains, plasmids and oligonucleotides used in this study.** Km^r^ - kanamycin resistance, DSMZ - Deutsche Sammlung von Mikroorganismen und Zellkulturen.

| Strain, oligonucleotides | Characteristics or sequence (5’-3’) | | Source or reference |
| --- | --- | --- | --- |
| Strain |  |  |  |
| *Sulfurospirillum multivorans*  Wild type |  |  | DSMZ 12446 |
| Oligonucleotides |  |  |  |
|  |  | Amplicon size (bp) |  |
| *hydB*_fw | GTT GAA GAT GCG CTT GGA | 234 | This work |
| *hydB*_rev | TTT GGG TTG CAA CAA GAT |  | This work |
| *hupL*_fw | GCG TTT GGA AGA GTT ATT GGA G | 214 | This work |
| *hupL*_rev | TAC GTA TTT GAC ATC CGC ACT C |  | This work |
| *echE*_fw | AGC GTT GAT GAC CCA GTT T | 249 | This work |
| *echE*_rev | ATA GCT CAA AAC GCC CAC |  | This work |
| *hyfG*_fw | TGA CGT GCC TCT AGG ACC TT | 205 | This work |
| *hyfG*_rev | CAT GGG CAT AAC CAC AGA TG |  | This work |
| *recA*_fw | TAA AGT GGC ACC TCC GTT TC | 266 | This work |
| *recA*_rev | CGC CAC ATG TCA TAA CCA TC |  | This work |
| *16S rRNA*_fw | GAG ACA CGG TCC AGA CTC CTA C | 255 | This work |
| *16S rRNA*_rev | CTC GAC TTG ATT TCC AGC CTA C |  | This work |

**Supplementary Table 2: Hydrogen oxidizing activity of *S. multivorans* crude extract with different electron acceptors.** Cells were grown on Pyr/PCE.

| Electron Acceptor | Redox potential (mV) | Wavelength (nm) | Specific activity (nkat mg^-1^) |
| --- | --- | --- | --- |
| Benzylviologen^1^ (BV) | -374 | 578 | 56.2 ± 5.2 |
| Methylviologen^1^ (MV) | -446 | 578 | 19.6 ± 4.6 |
| NAD^+2^ | -320 | 365 | <0.01 |
| Methylene blue^1^ (MB) | +11 | 570 | 8.6 ± 1.1 |
| Nitroblue tetrazoliumchloride^2^ (NBT) | +50 | 593 | <0.01 |
| Phenazine methosulfate^2^ (PMS) | +65 | 388 | <0.01 |

^1^Enzyme activity assays with BV, MV and MB are described in Materials & Methods

^2^NAD^+^, NBT and PMS were prepared in concentrations of 0.2 mM in 50 mM Tris-HCl (pH 8.0)
